# Physical Exercise Prevents the Cognitive Decline among Older Adults in Romania

**DOI:** 10.3390/healthcare12171791

**Published:** 2024-09-07

**Authors:** Andrei Ciobica, Romeo Dobrin, Alin Iordache, Ioannis Mavroudis, Cezar Honceriu, Antoneta Dacia Petroaie, Veronica Bild, Alexandru Vasincu, Răzvan-Nicolae Rusu, Alin Ciobica, Walther Bild

**Affiliations:** 1 Department of Physiology, “Grigore T. Popa” University of Medicine and Pharmacy, 16 Universitatii Street, 700115 Iasi, Romania; nicusor-andrei-ms-ciobica@d.umfiasi.ro (A.C.); walther.bild@umfiasi.ro (W.B.); 2 Department of Psychiatry, “Grigore T. Popa” University of Medicine and Pharmacy, 16 Universitatii Street, 700115 Iasi, Romania; petru.dobrin@umfiasi.ro; 3 Department of Neurosurgery, “Grigore T. Popa” University of Medicine and Pharmacy, 16 Universitatii Street, 700115 Iasi, Romania; alin.iordache@umfiasi.ro; 4 Department of Neurology, Leeds Teaching Hospitals, NHS Trust, Leeds LS2 9JT, UK; ioannis.mavroudis@gmail.com; 5 Department of Physiology, “Alexandru Ioan Cuza” University of Iasi, 20A Carol I Avenue, 700505 Iasi, Romania; chonceri@yahoo.fr; 6 Department of Family Medicine, “Grigore T. Popa” University of Medicine and Pharmacy, 16 Universitatii Street, 700115 Iasi, Romania; 7 Department of Pharmacodynamics and Clinical Pharmacy, “Grigore T. Popa” University of Medicine and Pharmacy, 16 Universitatii Street, 700115 Iasi, Romania; veronica.bild@umfiasi.ro (V.B.); razvan-nicolae.rusu@umfiasi.ro (R.-N.R.); 8 Center of Biomedical Research, Romanian Academy, Iasi Branch, 2 Teodor Codrescu Street, 700481 Iasi, Romania; alin.ciobica@uaic.ro; 9 Department of Biology, “Alexandru Ioan Cuza” University of Iasi, 11 Carol I Avenue, 700505 Iasi, Romania; 10 Academy of Romanian Scientists, 54 Splaiul Independentei, Sector 5, 050094 Bucharest, Romania; 11 “Ioan Haulica” Institute, Apollonia University, 11 Pacurari Street, 700511 Iasi, Romania

**Keywords:** physical exercise, cognitive decline, exercise intensity, exercise frequency, old adults, ageing population

## Abstract

Cognitive decline is one of the most important challenges related to the aging process, due to its important impact on individuals. Several studies have reported that physical exercise with a specific intensity and frequency is beneficial for maintaining cognitive health in the ageing population. The present study investigated the impact of general physical exercise on cognitive health in the older population in Romania. The study involved 60 individuals (60% male, 40% female), with a mean age of 60.78 years (SD = 2.97). The Health Interview Survey and The Minnesota Heart Survey assessed exercise frequency and intensity, while the Montreal Cognitive Assessment (MoCA) determined mild cognitive impairment (MCI) levels. The results of the statistical analysis showed that high-intensity physical exercise at a frequency of three to four times a week at the age of 40–50 years is recommended in order to significantly reduce cognitive decline. In addition, for the age of 60 years old, the results established that engaging in physical activities of a moderate intensity with a frequency of 2–3 times per month is sufficient to maintain healthy cognition. The findings suggest that exercise can serve as a behavioral intervention to mitigate cognitive dysfunction and complement past research on its cognitive health advantages.

## 1. Introduction

Ageing population is an irreversible worldwide trend which stems from a demographic transition towards living longer [1]. Although during the last decades of the 20th century, it was expected that human life expectancy would reach a plateau, it did not hit the predicted ceiling and continued to grow in a similar manner as before [2]. This increase in life expectancy is a sign of better health overall, with the number of years individuals live without severe disability, in relatively good health, being at high rates in many places. The World Health Organization estimates that one in six people in the world will be aged 60 years or over by 2030, and it could reach a number of 2.1 billion by 2050 [3].

One of the most important challenges related to the aging process refers to the cognitive impairment that can occur in individuals, a problem which is of such proportions that some professionals consider it ”the elephant in the room”. During the aging process, a certain level of cognitive decline can lead to mild cognitive impairment (MCI) and dementia [4]. On the scale of a cognitive continuum, with normal aging and dementia as reference points, MCI is in an intermediate position represented by cognitive disorders that do not significantly affect the social or occupational functionality of the individual [5].

There is immense interest in searching for the right approach that could enhance cognitive and brain function throughout the lifespan, which could lead to an improvement in aspects such as decision-making abilities, career opportunities, and overall quality of life. One of the most promising ways for positively influencing brain plasticity, cognitive function, and wellbeing and for reducing the risk of age-related cognitive decline seems to be physical exercise [6,7]. The mechanisms through which physical activity positively influences cognition are related to the body’s adaptations to enhance exercise performance, which are also beneficial for the brain. Some of these mechanisms involve the release of growth factors that contribute to neuronal development and growth, neuro- and angiogenesis, lactate production which provides energy to the brain, reducing neuroinflammation through the release of anti-inflammatory cytokines, and reducing oxidative stress through the increase in mitochondrial biogenesis and antioxidant enzyme activity as well as through the release of dopamine and serotonin, which modulate cognition and regulate neurogenesis [8]. Several studies have reported that physical exercise leads to mechanisms such as an increase in the volume of gray matter in the frontal and hippocampal regions, while also leading to reduced damage in the gray matter. A facilitated release of neurotrophic factors such as peripheral brain-derived neurotrophic factor, an increase of blood flow, improvement in cerebrovascular health, as well as benefits on lipid and glucose metabolism for brain nutrition have also been reported [6,7].

Although the terms “physical activity” and “physical exercise” are terms that describe different concepts, they are often confused in the literature. Therefore, it is preferable to distinguish between the two concepts. “Physical activity” refers to any movement of the human body produced by skeletal muscles, which results in an energy expenditure expressed in kilocalories and which includes a wide range of functional, leisure, and everyday activities [9]. On the other hand, the term “physical exercise” describes a form of physical activity that is planned, organized, and performed intentionally, having a specific and well-defined purpose. In turn, physical exercise can be of several types: aerobic exercise, resistance training, or a combination of the two [9].

Physical exercise such as walking, gardening, dancing, or stretching is considered light exercise, while activities such as hiking, aerobics, or martial arts are of moderate intensity. Vigorous or high-intensity exercises are considered ones such as jogging, cycling, climbing, tennis, or football.

In the present study, we analyze how general physical exercise influences cognitive health. Moreover, our experimental design allowed us to investigate whether the level of physical exercise, both in middle age (40–50 years) and in the last calendar year, influences the participants’ risk of cognitive decline. Studying the effects of physical exercise in these two distinct time frames will allow us to establish both the level of intensity and the frequency of physical exercise required for a maximum beneficial effect on general cognition.

We chose this research topic due to the extremely high socio-economic impact of dementia, involving direct costs per year, accounting for USD 23,796/patient worldwide and in Romania, somewhere around USD 5200 [10]. Thus, it is very important to diagnose MCI early and to uncover all the factors that could prevent or postpone the onset of this condition.

To our knowledge, this is one of the first studies that aims to assess the influence of general physical exercise on cognitive health in Romania.

## 2. Materials and Methods

Our sample consisted of 60 participants who underwent various treatments within the Socola Institute of Psychiatry Iasi, Romania. A total of 60 percent were males; 40%, females. The mean age for our group of participants was 60.78 years old (SD = 2.97). The oldest individual was 65 years old, while the youngest was 55.

The research was carried out on the basis of the ethics approval obtained from the Ethics Commission of the University of Medicine and Pharmacy “Grigore T. Popa” Iasi, No. 167/07.03.2022 and the opinion of the Ethics Committee 7637/06.04.2021 issued by the Socola Institute of Psychiatry Iasi.

### 2.1. Measurement of Physical Exercise

We measured the frequency and intensity of physical exercise using questions from two previously validated instruments, the translated versions of The Health Interview Survey and The Minnesota Heart Survey.

Firstly, participants were asked to directly provide information about physical exercise performed within the last calendar year. Secondly, they were asked about the physical exercise performed at the age of 40 to 50 years. Furthermore, the questionnaire asked about light, moderate, or vigorous exercise.

Light exercise has been defined as walking, gardening, stretching, and dancing. Moderate exercise has been defined as brisk walking, hiking, aerobics, swimming, tennis at doubles, small field football or other recreational team sports, moderate use of stationary and elliptical bicycles, yoga, martial arts, and activities that involve lifting moderate weights. Vigorous exercise has been defined as jogging, mountain hikes, climbing cycling, tennis, football or other intense team sports, intense use of fitness machines, skiing, and other alpine sports. 

For each category of intensity, a further investigation was carried out on the frequency of these physical activities (frequency per month or per week). Thus, 6 levels of exercise frequency were formed: 1. Once a month or less; 2. Two to three times a month; 3. One to two times a week; 4. Three to four times a week; 5. Five or six times a week; 6. Every day.

The questionnaires related to exercise intensity and frequency were measured for two distinct timelines. In the first phase, the intensity and frequency of exercise were measured when the participants were between 40 and 50 years of age (mid-life). Then, participants were directly asked about the intensity and frequency of physical exercise in the last calendar year. We decided on this experimental design in order to discover how the physical exercise performed in both mid-life and in the last calendar year influences MCI.

### 2.2. Montreal Cognitive Assessment—MoCA

MoCA has been developed as a tool for patients with MCI who usually have scores in the normal range when tested using the Mini-Mental State Examination (MMSE). The MoCA test is able to differentiate between normal cognition and MCI, as well as between MCI and early dementia [11].

The initial MoCA validation study reported a sensitivity of 100% and 87% specificity in detecting the early stage of Alzheimer’s disease (cut-off 26). The original study also reported a sensitivity of 90% in MCI detection [11].

MoCA has been translated into over 100 languages and dialects. Its validity has been established for the detection of cognitive impairment in various clinical populations diagnosed with Parkinson’s disease, cerebral metastases, and vascular accidents. The high sensitivity of MoCA to MCI detection and dementia has been confirmed by studies in several countries [12,13,14,15].

It covers eight areas of cognitive skills: visual-space functions, executive functions, short-term memory, attention, concentration, working memory, language, and orientation. This gives a maximum score of 30, and a score of over 26 points is considered normal. The duration of the test is about 10 min [11].

The research technique was oral (face-to-face), the tests being applied to individuals who underwent treatment at the Socola Institute of Psychiatry Iasi in a separate room after having been assured of the confidentiality of the discussion.

### 2.3. Statistical Analysis

For the statistical processing, the IBM SPSS Statistics 26.0 software was used. The statistical method used was One-Way ANOVA, and for the comparison of means of each experimental group we used the post hoc Fisher’s Least Significant Difference (LSD) analysis. The significance threshold was defined at *p* < 0.05. The confidence interval was 95%.

## 3. Results

### 3.1. The Intensity of Physical Exercise in the Mid-Life (40–50 Years)

**Figure 1:**
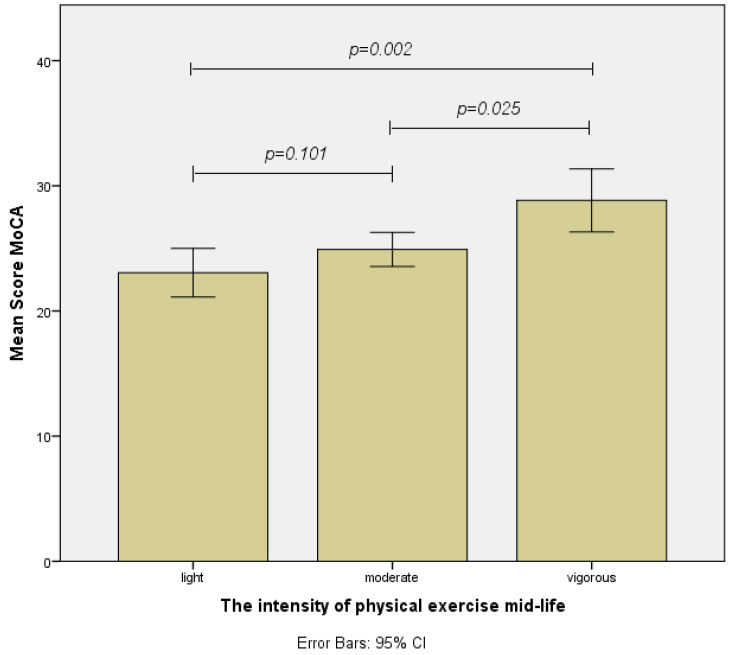
Differences regarding the total MoCA score for every intensity of physical exercise group at mid-life.

Our One-Way ANOVA demonstrated that there were significant differences between the three intensity groups F(2,57) = 5.094, *p* = 0.009. The LSD post hoc analysis showed that there were significant differences between the MoCA scores of individuals who participated in high-intensity exercise and those who participated in moderate exercise (MD = 3.917, *p* = 0.025). Moreover, significant differences were also observed between the high-intensity group and the light-intensity group (MD = 5.778, *p* = 0.002). On the other hand, no significant difference was observed between light- and moderate-intensity groups (MD = 1.861, *p* = 0.101) (Figure 1). A further analysis of statistically significant or non-significant differences between the three exercise intensities during middle age can be seen in Figure 1.

### 3.2. Frequency of Exercise at Mid-Life (40–50 Years)

The initial ANOVA analysis showed that there were significant differences between the six frequencies of physical exercise groups F(5,54) = 8.855, *p* < 0.001. The LSD post hoc analysis demonstrated that there were significant differences between the MoCA scores of individuals who participated in exercise at a frequency of once a month or less and the following frequency groups: three to four times a week (MD = 6.067, *p* < 0.001), five to six times a week (MD = 7, *p* = 0.001), and every day (MD = 7.5, *p* = 0.004). On the other hand, the group of individuals who participated in physical exercises with a frequency of once a month or less did not achieve significantly different MoCA total scores compared to groups with a frequency of two to three times a month (MD = 0.571, *p* = 0.656) or one to two times per week (MD = 2.111, *p* = 0.168).

In addition, significant differences were also observed between MoCA total scores of individuals who participated in physical exercise at a frequency of two to three times per month, and groups with a frequency of three to four times a week (MD = 5.495, *p* < 0.001), five–six times a week (MD = 6.429, *p* = 0.001), and every day (MD = 6.929, *p* = 0.005). However, a non-significant difference was reported between the group of participants with a physical exercise frequency of two to three times per month and the group who exercised once or twice a week (MD = 1.540, *p* = 0.233).

**Figure 2:**
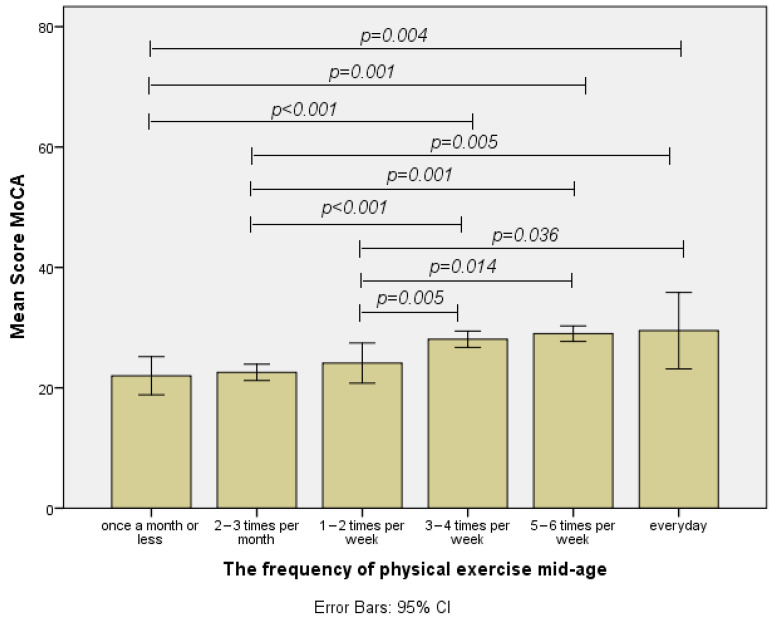
Differences regarding the MoCA total score for each frequency group of physical exercise at mid-life.

Moreover, significant differences were also observed between the total MoCA scores of individuals who participated in physical exercise at a frequency of once or twice a week and groups with a frequency of exercise of three to four times a week (MD = 3.956, *p* = 0.005), five–six times a week (MD = 4.889, *p* = 0.014), and every day (MD = 5.389, *p* = 0.036) (Figure 2).

From this point, no matter how much the frequency of exercise increased, there were no significant differences in the total score of MoCA between the groups: three to four times a week compared to five or six times per week (MD = 0.933, *p* = 0.607), three to four times per week compared with every day (MD = 1.433, *p* = 0.554), and the group with an exercise frequency of five to six times a week was not significantly different from the one with a daily exercise frequency (MD = 0.5, *p* = 0.858). A clearer view of statistically significant or non-significant differences between the six levels of exercise frequency during mid-age (40–50 years old) can be seen in Figure 2.

### 3.3. The Intensity of Exercise in the Last Year

**Figure 3:**
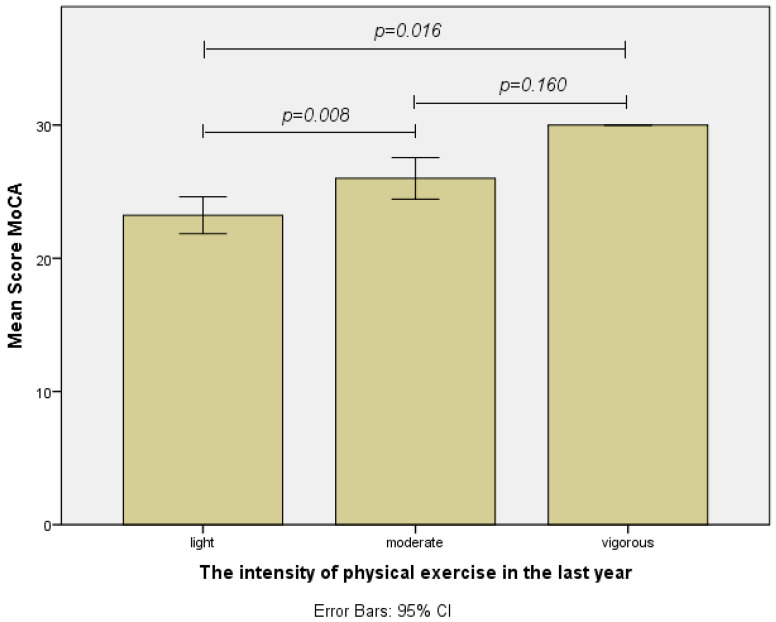
Differences regarding the MoCA total score by exercise intensity groups in the last year.

Regarding the last calendar year, the statistical analysis revealed that there were significant differences between the total MoCA scores of individuals from the three intensity groups F(2,57) = 5.700, *p* = 0.006. Furthermore, the post hoc analysis showed significant differences regarding the total MoCA score between individuals who participated in light exercise and those who participated in moderate exercise (MD = 2.676, *p* = 0.008). Moreover, significant differences were observed between the light-intensity group and the high-intensity group (MD = 7.676, *p* = 0.019). This time, no significant difference was observed between the high-intensity and moderate-intensity groups (MD = 4, *p* = 0.160) (Figure 3).

Furthermore, a more evident view of the statistically significant or non-significant differences between the three physical exercise intensities in the last calendar year can be seen in Figure 3.

### 3.4. The Frequency of Exercise in the Last Year

**Figure 4:**
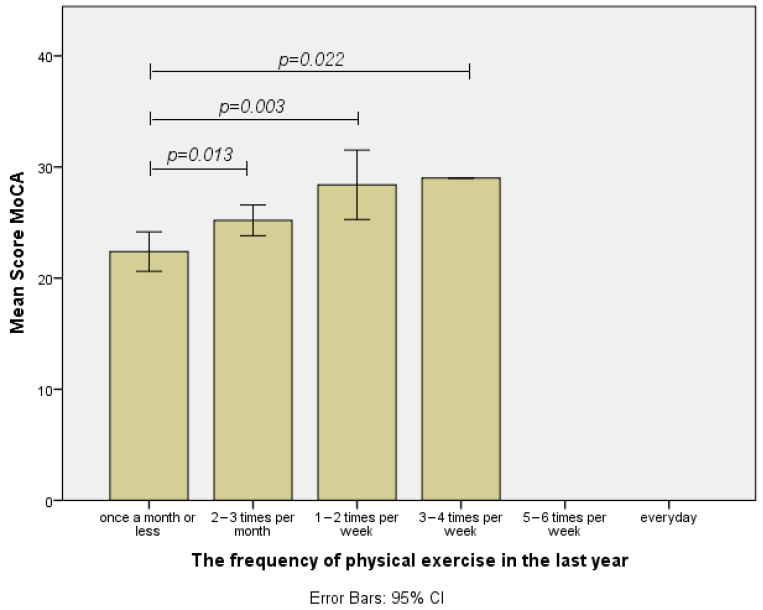
Differences regarding the MoCA total score by exercise frequency groups in the last year.

Before describing the results for this category, it should be noted that, for this timeline, no individual reported a physical exercise frequency of five to six times a week or each day. Therefore, the initial One-Way ANOVA was significant F(3,56) = 4.922, *p* = 0.004. Furthermore, the LSD post hoc analysis showed that there were significant differences between the total MoCA scores of individuals who participated in physical exercises at a frequency of once a month or less and the following groups of frequencies: two to three times a month (MD = 2.811, *p* = 0.013), once or twice a week (MD = 6.011, *p* = 0.003), and three to four times a week (MD = 6.611, *p* = 0.022). On the other hand, the group of individuals who participated in physical exercises two or three times a month did not obtain significantly different total scores from the groups with a frequency of once or twice a week (MD = 3.2, *p* = 0.081) or three to four times week (MD = 3.8, *p* = 0.171) (Figure 4). Moreover, non-significant differences were reported between the group of participants with a physical exercise frequency of one to two times a week and the group with a frequency of three to four times a week (MD = 0.6, *p* = 0.850). For a clearer analysis of statistically significant or non-significant differences between the four levels of exercise frequency in the last calendar year, Figure 4 can be consulted.

## 4. Discussion

The importance of the present study comes from its attempt to validate the hypothesis that an individual’s physical activity level directly affects one’s risk of developing cognitive disorders such as MCI, or even dementia or Alzheimer’s disease. We chose to measure the frequency and intensity of physical exercise in two periods of life, at mid-life and 40–50 years old, but also the intensity and frequency of physical exercise in the last calendar year. For the intensity of physical exercise, we had three levels: light, moderate, and vigorous. In addition, for the frequency of physical exercise, we had six levels: once a month or less, two or three times a month, once or twice a week, three or four times a week, five to six times a week, and every day.

Regarding the intensity of physical exercise in mid-life (40–50 years old), the results of our study showed that only participants who engaged in high-intensity physical activities achieved significantly higher total scores on the MoCA test, compared to the participants engaging in moderate or light physical exercise. No statistically significant difference was found between the moderate-intensity group and the light-intensity group. Hence, it can be concluded that exercise performed at mid-life (40–50 years old) should be at high intensity to positively influence the preservation of cognitive abilities later in life. Furthermore, physical exercise with a moderate intensity does not seem to be sufficient to positively influence cognitive decline. Choe et al. performed a study that measured the associations of midlife-initiated walking intensity with general cognition which reported similar results. According to the authors, the midlife-initiated walking intensity was significantly associated with better episodic memory and overall cognition. In addition, the same authors reported that there was no difference in overall cognition performance according to the walking duration of the participants [16]. 

Furthermore, the results of our study regarding the frequency of physical exercise for the mid-life (40–50 years old) demonstrated that participants who engaged in physical exercise once a month or less achieved significantly lower total scores at MoCA compared to those with a frequency of three to four times a week, to those with a frequency of five to six times per week, or to participants with a physical exercise frequency of every day. No significant difference in the total score of MoCA was found between participants with the frequency of once a month or less and the frequencies of two or three times a month or once or twice a week. In fact, this was the trend observed for all of the first three frequency levels (once a month or less, two to three times a month, and once or twice a week); compared to each other, no significant difference in total MoCA scores was found; however, any other possible pair with the last three exercise frequency levels (three to four times a week, five to six times a week, and every day) resulted in a statistically significant difference regarding the total score of the MoCA test; individuals with a lower physical exercise frequency obtained lower total scores on the MoCA. Therefore, the results of our statistical analysis for this section are conclusive. The exercise frequency threshold required to influence the total scores on the MoCA is located between one to two times a week and three to four times a week. Hence, it can be concluded that a minimum exercise frequency of three to four times a week at the age of 40–50 years old is necessary to prevent cognitive decline at a later age. 

Therefore, the results of this study recommend high-intensity physical exercise and a frequency of three to four times a week at the age of 40–50 years old to significantly reduce cognitive decline and the chance of developing MCI and later dementia.

In regard to the intensity of physical exercises performed by the participants in the last calendar year, the results revealed by our statistical analysis are also extremely interesting. In this particular case, there were significant differences regarding the total MoCA scores between the groups reporting a light intensity and the group reporting moderate exercise intensity, and also between the light- and the high-intensity groups. The interesting part of these results was the lack of significant difference in total MoCA scores between the group that participated in moderate physical activities and those who participated in high-intensity physical activities. Unlike the results for the intensity of mid-life exercise, for older age (in the last calendar year), a moderate intensity is sufficient to positively influence total MoCA scores. Moreover, increasing exercise intensity at this age range from moderate to high does not significantly influence the MoCA score. These results are in concordance with various studies who reported that in older adults (60 years old or older), moderate-intensity physical exercise was higher associated with positive results on all cognitive outcomes in comparison with vigorous-intensity physical exercise [17].

Lastly, the results regarding the last calendar year exercise frequency’s influence on total MoCA scores are again different from those reported for the middle-aged. If for the 40–50 years old interval, the minimum frequency necessary to influence the observed MoCA score was three to four times a week, for the last calendar year, a frequency of two to three times a month is sufficient to observe a significant positive difference in the total MoCA scores. Moreover, participants who reported a greater exercise frequency than two or three times a month did not score significantly higher in the MoCA test compared to the participants with a frequency of two or three times a month. Consequently, it can be concluded that for the age of about 60 years (the average age of our participants), moderate exercise is recommended at a frequency of 2–3 times per month in order to maintain healthy cognition. The available scientific literature supports the conclusion that later in life, there is no need for a vigorous intensity or a higher frequency of physical exercise. For example, a recent study conducted by Yu et al. investigated the effects of 12-week walking exercise interventions at various frequencies and intensities on improving cognitive performance in older adults (mean age = 63 years old). The results of this research demonstrated that the moderate and vigorous intensities at once-a-week and three-times-per-week frequencies had similar effects on improving global cognitive performance [18].

Therefore, in addition to pharmaceutical treatment, exercise is a good example of a behavioral intervention that could play an important role in treating mild cognitive dysfunction. Physical exercise may be potentially effective because studies in this area suggest that it could improve cognition by increasing neurogenesis and synaptogenesis and reducing the accumulation of beta amyloid in the brain [19].

The beneficial effects of physical exercise on cognition in general have been demonstrated both in animal models and in clinical trials that had older participants [20,21]. These studies highlighted the positive effect of regular exercise on the survival and functioning of neurons, neuro-inflammation, vascularization, neuroendocrine responses to stress, and reduction in beta amyloid accumulation [22,23,24].

On the other hand, regular exercise has been demonstrated to have a significant positive effect on physiological processes such as blood glucose regulation or cardiovascular health [25]. It is noteworthy to consider that possible disorders of these processes are shown to be important risk factors in MCI and dementia [25,26]. Moreover, the benefits of exercise also extend to cognitive processes that suffer important dysfunctions in patients suffering from MCI. These processes are selective attention, working memory, the ability to perform multiple tasks, planning ability, and organizational capacity [27].

Because MCI is seen as a precursor of Alzheimer’s disease, many studies have focused their attention on the benefits of aerobic exercise in this condition. For example, Baker et al. examined the possible positive effect of aerobic exercise on individuals with MCI. The results reported improvements in different areas of cognition, such as the ability to perform multiple tasks at the same time, cognitive flexibility, selective attention, and the efficiency of information processing. Furthermore, other improvements were found in glucose metabolism, in the cardiorespiratory system’s health, and in a significant reduction in adipose tissue [28].

Although the optimal dose of physical exercise, which refers to its intensity, frequency, and duration, has not yet been found, positive relationships between high intensity and cognitive health have been reported in older populations. In a meta-analysis of 16 studies, the risk for dementia and Alzheimer’s disease was the lowest among participants with a high level of physical activity. These results suggest that physical exercise may have a protective effect and it may reduce the risk of dementia by 28% and the risk of Alzheimer’s disease by 45% [29].

There are various biomolecular mechanisms that may explain the observed benefits of physical exercise on cognitive outcomes. Firstly, it is widely known that physical exercise significantly improves the management of cardiovascular risk factors (such as diabetes, hypertension, dyslipidemia, and obesity) that are traditionally associated with poor cognitive performance [30].

Secondly, the scientific literature reports that physical exercise can increase neurogenesis and synaptic plasticity [30]. Moreover, physical activity, especially aerobic exercise, is associated with increased brain-derived neurotrophic factor, a factor that can stimulate neuronal cell growth and keep neurons healthy [31].

Thirdly, using neuroimaging techniques, additional evidence for the impact of physical exercise on brain function and structure has been reported [32,33,34]. Specifically, physical exercise is linked to increased brain volumes in areas critical for memory and information processing [35]. Furthermore, higher physical activity was linked with greater brain volume, which in turn was found to reduce the risk of cognitive impairment and the risk of dementia [36].

In addition, physical exercise interventions have an important role in improving several non-cognitive markers such as degree of disability, incidence and prevalence of falls, and various neuropsychiatric symptoms in individuals with dementia [37]. All these results are of significant clinical importance due to a high level of co-occurrence and multi-morbidity.

## 5. Conclusions

Our findings suggest that a minimum frequency of physical exercise of three to four times a week is necessary during middle age to reduce the risk of cognitive decline, of developing MCI, as well as of developing dementia later in life; while for older adults, normal cognition can be maintained with moderate-intensity exercise at a frequency of two to three times a month. Therefore, exercise is a relevant behavioral intervention that can be implemented to mitigate cognitive dysfunction due to its important cognitive health advantages.

## Data Availability

The datasets used and/or analyzed during the current study are available from the corresponding author upon reasonable request.

## Abbreviations

LSD	Least Significant Difference
MCI	Mild cognitive impairment
MMSE	Mini-Mental State Examination
MoCA	Montreal Cognitive Assessment
